# Diabetes treatment and hypoglycaemic episodes in elderly patients at nursing homes in Uppsala County

**DOI:** 10.1080/03009734.2016.1198441

**Published:** 2016-06-29

**Authors:** Angelica Walfridsson, Maja Sehlberg, Ulrika Gillespie, Jonathan Dahlkvist, Hans-Erik Johansson

**Affiliations:** a Östervåla Primary Health Care Centre, Östervåla, Sweden;; b Uppsala University, Faculty of Biomedicine, Uppsala, Sweden;; c Uppsala University Hospital (Akademiska Sjukhuset), Uppsala, Sweden;; d Uppsala Läns Landsting, Uppsala, Sweden;; e Department of Public Health and Caring Sciences/Geriatrics, Uppsala University, Uppsala, Sweden

**Keywords:** Diabetes medication, elderly patients, HbA1c, hypoglycaemia, nursing homes

## Abstract

**Aim:**

The aim of this study was to examine the situation for elderly patients with diabetes living in nursing homes with regard to diabetes treatment, clinical variables, and vascular complications associated with diabetes. A second aim was to evaluate if the patients were at risk of hypoglycaemia.

**Methods:**

This was a cross-sectional study including diabetes patients from all 30 nursing homes in Uppsala County, Sweden. Current antidiabetic medications, HbA_1c_, hypoglycaemic events, and diabetes complications were registered from the medical records. The patients were stratified into a general group and divided into three groups according to HbA_1c_ (<52, 52–73, and >73 mmol/mol).

**Results:**

Of 1,350 individuals, 218 patients were identified with diabetes mellitus. The diabetes duration was 11.2 ± 9.4 years and their serum HbA_1c_ concentration 56.0 ± 1.2 mmol/mol. Hypoglycaemic events were reported in 24% of the diabetic individuals, and 43.1% of them had HbA_1c_ <52 mmol/mol (mean value 44.0 ± 1.1 mmol/mol). Of these, 36% were taking antidiabetic drugs. Another 35.8% of the patients had HbA_1c_ values between 52–73 mmol/mol (mean value 60.0 ± 1.1 mmol/mol), and 82% of these patients were taking antidiabetic drugs. Almost 80% of the diabetic patients had either micro- or macrovascular complications, with diabetes duration as an association for both micro- or macrovascular complications and hypoglycaemic events.

**Conclusions:**

A reduction of the use of antidiabetic drugs with follow-up of HbA_1c_ level should be considered, especially for multimorbid elderly patients with low HbA_1c_ and hypoglycaemia.

## Introduction

The prevalence of diabetes is increasing worldwide, affecting more than 8% of the adult population. Diabetes is one of the leading causes of cardiovascular disease and death (1–3). In general, it is recommended that patients with type 2 diabetes mellitus (T2DM) receive intensive therapy with tight glycaemic control to avoid micro- and macrovascular complications (4,5). The value of an intensive diabetes therapy for elderly patients, especially with comorbidities, is under much discussion, and recent guidelines are emphasizing avoidance of very tight glucose control and thereby hypoglycaemia (6–8). Many of the elderly patients have a limited life expectancy, and studies have shown intensive plasma glucose-lowering treatment to have poor or no effect on the prevention of micro- and macrovascular complications, especially if the diabetes duration is longer than 8–10 years (9,10).

Due to the complexity of ageing and declining body functions, often associated with multiple chronic illnesses, e.g. dementia and polypharmacy, these patients run a greater risk of severe hypoglycaemic complications (11,12). Hypoglycaemic events may cause emotional stress and also cognitive impairment, cardiac arrhythmias, and even death (13). The main aim of this study was to examine the situation for elderly patients with diabetes in all 30 nursing homes in Uppsala County with regard to HbA_1c_ values, diabetes treatment, and micro- and macrovascular complications. Patients were divided into three groups according to HbA_1c_ value (<52, 52–73, and >73 mmol/mol). A second aim was to examine differences in diabetes treatment between the groups and the risk for excessive treatment and hypoglycaemia.

## Subjects and methods

### Patients and data collection

**Figure 1.**
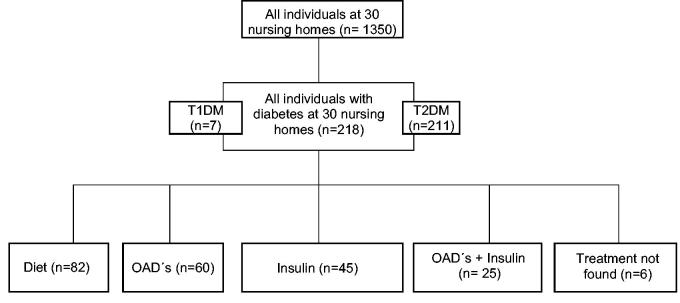
Design of the recruitment of patients.

In 2012 we collected a list of 1,350 patients from 30 different nursing homes in Uppsala County, Sweden (Figure 1). Uppsala is the capital of Uppsala County and the fourth largest city in Sweden. Uppsala County has more than 200,000 inhabitants. Data were collected from the patients’ medical records in Electronic Medical Records System (Cosmic), and medication details were collected from Computer Based Medication System (Pascal). We identified all patients with diabetes, both type 1 diabetes mellitus (T1DM) and T2DM. From the patients’ medical files we registered last known HbA_1c_ value, diabetes duration, age, gender, weight, height, plasma creatinine, current antidiabetic medications, micro- and macrovascular complications, and hypoglycaemic episodes. Retinopathy, nephropathy, and neuropathy were designated as microvascular complications, while myocardial infarction, unstable angina pectoris, brain haemorrhage, brain infarction, and transient ischemic attack were designated as macrovascular complications.

Hypoglycaemic events were defined as plasma blood glucose <4 mmol/L. We categorized hypoglycaemic events as severe or minor events. Events leading to hospitalization, events that needed treatment in the emergency room, and events emerging during hospital care were categorized as severe events. Hypoglycaemic events only noted in a patient’s medical journal were considered as minor events. The patients were first stratified into a general group and then divided into three groups according to HbA_1c_ (<52, 52–73, and >73 mmol/mol). Statistical analyses were made to examine the differences between the groups in antidiabetic treatment, medical dosages, and hypoglycaemic complications.

Patients on oral antidiabetic drugs (OADs) as well as rapid-acting insulin when needed were recorded as OADs. Patients on diet treatment with rapid-acting insulin when needed were recorded as diet treatment.

### Ethics

The study was approved by the regional ethics review board at Uppsala University.

### Statistics

Results are given as arithmetic means with their standard deviations. Comparisons were made using chi-square tests with the Yates correction factor for analyses in differences between diabetes treatment and hypoglycaemic episodes within the groups. The differences between medication doses were analysed using independent samples *t* test. To avoid too small subgroups a cut-off level at HbA_1c_ 52 mmol/mol was chosen. A *P* value <0.05 was considered statistically significant. Multivariable analyses taking co-morbidities was not performed. Findings of association of HbA_1c_ with risk of hypoglycaemia are crude, and they are not adjusted for the influence of differences in other variables, such as age, diabetes duration, or gender. A subgroup analysis on patients on pharmacological treatment for diabetes was performed. Statistical software JMP 5.0 for PC (SAS Corporation, Cary, TX, USA) was used.

## Results

**Figure 2.**
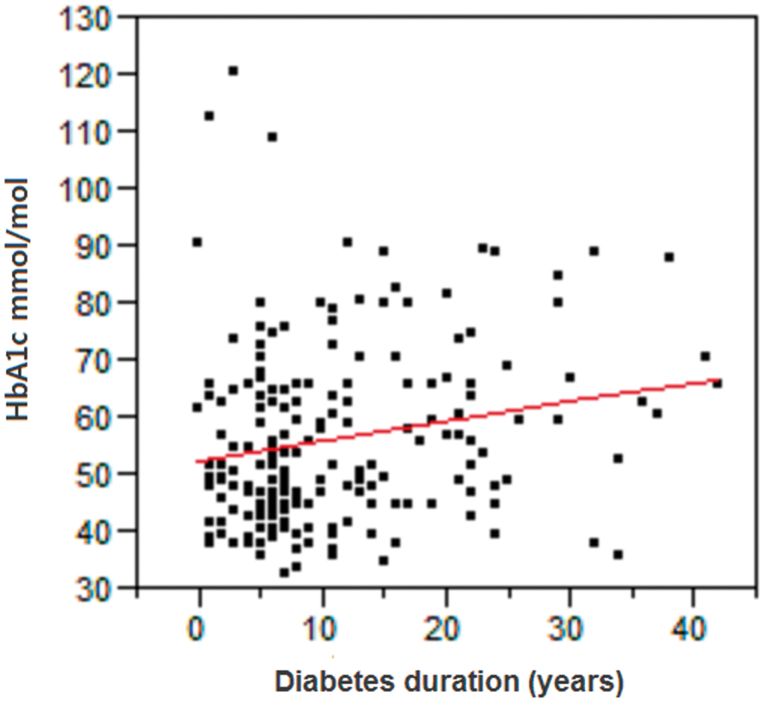
The correlation between HbA_1c_ (mmol/mol) and diabetes duration (years); *P =* 0.006.

Of 1,350 individuals at 30 nursing homes, 218 patients (137 women, 81 men) were identified with diabetes mellitus (16.1%) (Table 1). Their mean BMI was 26.3 ± 5.7 kg/m^2^, and mean age was 84.6 ± 8.0 years. Seven patients had T1DM, and 211 patients had T2DM. Mean HbA_1c_ was 56.0 ± 1.2 mmol/mol (data missing for 18 patients), and average diabetes duration was 11.2 ± 9.4 years. The diabetes duration in the different HbA_1c_ groups was 8.6 ± 1.4, 13.0 ± 11.0, and 16.8 ± 10.7 years, respectively. Longer diabetes duration was correlated with higher HbA_1c,_ (*P* = 0.006) (Figure 2). The creatinine clearance value was 53.9 ± 26.9 mL/min.

**Table 1.** Clinical characteristics of 218 diabetes patients at 30 nursing homes, and type of diabetes treatment. Patients were divided into three groups by their different HbA_1c_ levels.

Groups	All	HbA_1c_ <52	HbA_1c_ 52–73	HbA_1c_ >73	No HbA_1c_
Number of patients	218	94	78	28	18
Gender (female/male)	137/81	59/35	50/28	17/11	11/7
Age (years)	84.6 ± 8.0	84.1 ± 7.8	84.3 ± 9.0	85.0 ± 7.4	
BMI (kg/m^2^)	26.3 ± 5.7	26.8 ± 5.8	26.8 ± 6.0	25.2 ± 5.1	
Duration (years)	11.2 ± 9.4	8.6 ± 6.4	13.0 ± 11.0	16.8 ± 10.7	
HbA_1c_ (mmol/mol)	56.0 ± 1.2	44.0 ± 1.1	60.0 ± 1.1	86.0 ± 1.2	
Creatinine	53.9 ± 6.9	57.0 ± 28.7	54.4 ± 25.9	52.3 ± 27.0	
Diabetes treatment:					
Unknown	6	3	2	1	
Dietary (*n*)	82	57^a^	12	2	11
OADs (*n*)	61	25	27	5	4
Insulin (*n*)	44	4^a^	25	12	3
OADs + insulin (*n*)	25	5^a^	12	8	0

Data shown are arithmetic means (±SD).

a Statistically significant difference (*P <* 0.05) between groups HbA_1c_ <52 and HbA_1c_ ≥52 mmol/mol.

BMI: body mass index; OADs: oral antidiabetic drugs.

Eighty-two patients (37.6%) were on diet treatment, 60 patients (27.5%) were on OADs, 44 patients (20.6%) had insulin treatment, and 25 patients (11.5%) had combined OADs + insulin treatment. The number of patients with diet treatment was higher (*P* < 0.05) in the group with HbA_1c_ <52 mmol/mol compared to patients with HbA_1c_ ≥52 mmol/mol. There were no differences between the groups (<52 and ≥52 mmol/mol), comparing numbers of patients being treated with only OADs (*P* = 0.80). The number of patients with insulin treatment and HbA_1c_ ≥52 mmol/mol was higher (*P* < 0.05) compared to patients with HbA_1c_ <52 mmol/mol. Also, the number of patients with combined OADs + insulin treatment was higher (*P* < 0.05) for patients with HbA_1c_ ≥52 mmol/mol than for patients with HbA_1c_ <52 mmol/mol.

Of a total of 69 patients on insulin treatment, 9 patients had HbA_1c_ <52 mmol/mol, and 54 patients had HbA_1c_ ≥52 mmol/mol (Table 2). For three patients HbA_1c_ data were missing, and all of them were treated with an insulin mixture, while two of them were also on intermediate-acting insulin. Daily doses of insulin were the same in all groups (*P* = 0.319) (Table 2). Moreover, there were no differences between the groups regarding daily doses of long-acting insulin (*P* = 0.365), intermediate-acting insulin (*P* = 0.271), mixed insulin (*P* = 0.490), and continuous rapid-acting insulin (*P* = 0.490).

**Table 2.** Patients treated with insulin as single therapy or in combination with oral antidiabetic drugs. Daily doses expressed in international units. The patients were divided into three groups by different HbA_1c_ levels.

Groups	All	HbA_1c_ <52	HbA_1c_ 52–73	HbA_1c_ >73
Number of patients (daily doses, IU):				
Insulin	69 (31 ± 21)	9 (33 ± 21)	37 (26 ± 19)	20 (35 ± 21)
Long-acting	14 (28 ± 18)	2 (37 ± 35)	7 (28 ± 21)	5 (24 ± 5)
Intermediate-acting	26 (20 ± 12)	3 (23 ± 11)	15 (16 ± 11)	6 (24 ± 13)
Mixtures	39 (30 ± 19)	5 (31 ± 17)	18 (24 ± 18)	13 (39 ± 21)
Rapid-acting	3 (17 ± 8)	1 (8 ± 0)	2 (22 ± 0)	0

Data shown are arithmetic means (±SD). Of a total of 69 patients with insulin treatment, 9 patients had HbA_1c_ <52 mmol/mol and 54 patients HbA_1c_ >52 mmol/mol. For three patients HbA_1c_ data were missing, and all of them were given an insulin mixture, while two of them were also on intermediate-acting insulin. No differences were observed between the groups regarding daily doses of insulin (*P =* 0.319).

IU: international units of insulin.

Hypoglycaemic events were observed in 52 patients (Table 3). A total of 106 hypoglycaemic events were identified. Thirty-four patients had severe hypoglycaemic events. Ten patients needed hospitalization due to severe hypoglycaemia, eight patients needed to attend the emergency room for treatment, and 16 patients had hypoglycaemic episodes emerging during hospital care while being treated for other reasons. There were notes about low plasma glucose concentrations, <4 mmol/L, in the medical records of 33 patients. For the sake of clarity it has to be mentioned that some patients were diagnosed with hypoglycaemia more than once. The number of patients treated for hypoglycaemic events was lower for patients in the group with HbA_1c_ <52 mmol/mol compared to groups with HbA_1c_ ≥52 mmol/mol. However, there was no significant difference between the groups <52 and ≥52 mmol/mol regarding hospitalization due to hypoglycaemic events.

**Table 3.** Patients with diabetes vascular complications and hypoglycaemic episodes. The patients were divided into three groups by different HbA_1c_ levels.

Groups	All	HbA_1c_ <52	HbA_1c_ 52–73	HbA_1c_ >73
Number of patients	218	94	78	28
Hypoglycaemia:				
Patients with no hypoglycaemic events, *n* (%)	166 (76.1)	82 (87.2)^a^	54 (69.2)	17 (60.7)
Patients with hypoglycaemic events, *n* (%)	52 (23.9)	12 (12.8)^a^	24 (30.8)	11 (39.3)
Severe hypoglycaemic events:				
Hypoglycaemia, hospitalization	10 (4.6)	3 (3.2)	2 (2.6)	5 (17.9)
Hypoglycaemia, emergency room	8 (3.7)	0	6 (7.7)	1 (3.6)
Hypoglycaemia, during hospital care	16 (7.3)	4 (4.3)	6 (7.7)	5 (17.9)
Minor hypoglycaemic events:				
Hypoglycaemia, notes in medical records	33 (15.1)	7 (7.4)	17 (21.8)	8 (28.6)
Vascular complications:				
No diabetes complications, *n* (%)	49 (22.5)	27 (28.7)	14 (17.9)	3 (10.7)
Complications from diabetes, *n* (%)	169 (77.5)	67 (71.3)	64 (82.1)	25 (89.3)
Microvascular complications, *n* (%)	45 (20.6)	14 (14.9)	17 (21.8)	10 (35.7)
Macrovascular complications, *n* (%)	74 (33.9)	38 (40.4)	25 (32.1)	7 (25.0)
Micro- and macrovascular complications, *n* (%)	50 (22.9)	15 (16.0)	22 (28.2)	8 (28.6)

Microvascular complications: retinopathy, nephropathy, neuropathy; macrovascular complication: myocardial infarction, instable angina pectoris, brain bleeding, brain infarction, TIA; hypoglycaemic episodes: plasma glucose <4 mmol/L.

a Statistically significant difference (*P <* 0.05) between groups HbA_1c_ <52 and HbA_1c_ ≥52 mmol/mol. There were no hypoglycaemic episodes reported for patients with diet treatment only, except for two patients during hospital care.

A separate analysis of patients receiving glucose-lowering pharmacological treatment (*n =* 130) showed the same pattern regarding hypoglycaemic events as in the general group (Table 3), with the exception that hypoglycaemia treated in the emergency room was more frequent in the group with HbA_1c_ ≥52 mmol/mol (Table 4). Further, 49 patients received insulin on demand, and these patients were involved in 50% of all major or minor hypoglycaemic episodes. Insulin-treated patients with hypoglycaemic episodes in the groups were 5/9, 27/37, and 18/20, respectively. Not surprisingly, there were no hypoglycaemic episodes reported for patients with diet treatment only, except for two patients during hospital care.

**Table 4.** Hypoglycaemic episodes in subsets of patients given glucose-lowering pharmacological treatment. The patients were divided into three groups by different HbA_1c_ levels.

Groups	All	HbA_1c_ <52	HbA_1c_ 52–73	HbA_1c_ >73
Number of patients	130	34	64	25
Hypoglycaemia:				
Patients with no hypoglycaemic events, *n* (%)	80 (61.5)	24 (70.6)^a^	40 (62.5)	14 (56.0)
Patients with hypoglycaemic events, *n* (%)	50 (38.5)	10 (29.4)^a^	24 (37.5)	11 (44.0)
Severe hypoglycaemic events:				
Hypoglycaemia, hospitalization, *n* (%)	9 (6.9)	2 (5.9)	2 (3.1)	5 (20.0)
Hypoglycaemia, emergency room, *n* (%)	8 (6.2)	0^a^	6 (9.4)	1 (4.0)
Hypoglycaemia, during hospital care, *n* (%)	15 (11.5)	3 (8.8)	6 (9.4)	5 (20.0)
Minor hypoglycaemic events:				
Hypoglycaemia, notes in medical records, *n* (%)	33 (25.4)	7 (20.6)^a^	17 (26.6)	8 (32.0)

Hypoglycaemic episodes: plasma glucose <4 mmol/L.

a Statistically significant difference (*P <* 0.05) between groups HbA_1c_ <52 and HbA_1c_ ≥52 mmol/mol.

Diabetic vascular complications were registered for 169 patients (Table 3). Of them, 45 patients had microvascular complications, 74 patients (33.9%) had macrovascular complications, and 50 patients had both micro- and macrovascular complications. Regarding vascular complications there were no statistically significant differences between the groups.

## Discussion

This cross-sectional study showed that diet treatment was most frequent in the group with HbA_1c_ <52 mmol/mol, and this group had also fewer hypoglycaemic complications. Insulin treatment was less frequent in this group, but OADs use was surprisingly equal in all groups. Diabetes duration was strongly correlated with higher HbA_1c_. A relatively large subgroup with HbA_1c_ <52 mmol/mol received pharmacological treatment: 40% had OADs and 10% insulin. With age, limited life expectancy, and risk of severe hypoglycaemia taken into consideration, the perspective is that there are no studies showing beneficial effects from tight glycaemic control among these patients (9,10,12,14). A reduction of hypoglycaemic drugs should therefore be considered for this group, with follow-up to find out if similar HbA_1c_ levels may still persist. If it proves difficult to maintain a lower HbA_1c_ level because of increased risk of severe hypoglycaemia or other complications, an HbA_1c_ level up to 70 mmol/mol may be recommended for these multimorbid elderly patients.

For patients in the group HbA_1c_ 52–73 mmol/mol, more than 80% of the patients received antidiabetic drug treatment, and 50% had insulin treatment. Insulin and sulfonylureas are associated with a high risk of hypoglycaemia (7). Fifty patients were treated with sulfonylureas, and 13 of them were found in the group with HbA_1c_ <52 mmol/mol. Further, with higher HbA_1c_ there was an increasing number of insulin-treated patients with hypoglycaemic episodes (56%, 73%, and 90%) in the three different HbA_1c_ groups. We could not find any differences regarding daily dosage/doses of insulin between the groups with different HbA_1c_ levels. This finding might indicate excessive treatment, especially for the patients in the group with HbA_1c_ <52 mmol/mol.

The results in this study are in accordance with findings from a previous study in nursing homes in Sweden (15). It was shown that a significant reduction of diabetic medication could be safely administered without increasing the risk for hyperglycaemia. That study was performed at nursing homes with a similar patient population (*n =* 98), and the diabetes prevalence was 15%, with a mean HbA_1c_ value of 57.0 ± 1.2 mmol/mol, and most patients were on antidiabetic drug treatment. Several international studies have evaluated intensive diabetes treatment in T2DM patients regarding complications and that there were negligible effects on preventing micro- and macrovascular complications, particularly after a longer diabetes duration of 8–10 years (9,10,14). In the present study the patients had a diabetes duration of almost 12 years. The Veterans Affairs Diabetes Trial has shown that, for patients with similar diabetes duration and when HbA1c was lowered to 51 mmol/mol, there was no beneficial effect on preventing diabetes complications (14). Studies on elderly T2DM patients have also indicated a risk for negative side effects, such as hypoglycaemia, of tight glycaemic control (12,15,16). Hypoglycaemic events have been shown to be frequent among elderly patients with T2DM and may cause brain damage, cognitive impairment, cardiac arrhythmias, and even death (13,17). Hypoglycaemia might also mimic cognitive disorders, e.g. confusion and anxiety, as well as other conditions, and cause unnecessary medication of the T2DM patients with sedatives and psychotropic drugs. Thus, withdrawal of diabetes medication in the elderly receiving tight glycaemic control is warranted, and it has been shown to be safe and that it decreases the risk of hypoglycaemia (15,16).

In this present study we found a total of 106 hypoglycaemic episodes in 52 patients. Compared to observations in previous studies, hypoglycaemic events were less frequent, which may indicate that the method for finding hypoglycaemic events was insufficient. It could not be excluded that episodes of hypoglycaemia were not reported, since hypoglycaemia may mimic several other conditions and, furthermore, many hypoglycaemic episodes are nocturnal.

The hypoglycaemic events tended to be more severe among patients with higher HbA_1c_, which might reflect a diabetes disease with poor glycaemic control (Table 3). The ACCORD study and other epidemiological studies have shown increased risk of death with high HbA_1c_ levels, but the patients could still not benefit from intensive glucose-lowering therapy. The rate of death was even higher among patients with more intensive glucose-lowering treatment (5,9,18).

Further, in our study, 49 patients received insulin on demand, and they were involved in 50% of all major hypoglycaemic episodes and 50% of all minor hypoglycaemic episodes. The fact that 130 patients received pharmacological treatment shows that the subgroup given insulin on demand more often suffered from hypoglycaemic episodes. Insulin treatment was more common among patients with HbA_1c_ ≥52 mmol/mol. This could possibly, to some extent, explain why hypoglycaemia occurred more frequently with increasing HbA_1c_. Longer diabetes duration was correlated with higher HbA_1c_, and these parameters were associated with increased risk for hypoglycaemic episodes and microvascular complications. Almost 80% of the diabetes patients had either micro- or macrovascular complications, and diabetes duration was associated with both hypoglycaemic episodes and micro- or macrovascular complications.

The results from this study suggest that multimorbid elderly patients with low HbA_1c_ suffering from hypoglycaemic episodes could benefit from a re-evaluation of their pharmacological treatments, including changes of agents used, dose reductions, or even stopping selected treatments (15). HbA_1c_ should be monitored quite frequently to ensure that serum HbA_1c_ concentrations are kept within reasonable levels.
